# Evolution of Evolvability in Gene Regulatory Networks

**DOI:** 10.1371/journal.pcbi.1000112

**Published:** 2008-07-11

**Authors:** Anton Crombach, Paulien Hogeweg

**Affiliations:** Theoretical Biology and Bioinformatics Group, Utrecht University, The Netherlands; University of Washington, United States of America

## Abstract

Gene regulatory networks are perhaps the most important organizational level in the cell where signals from the cell state and the outside environment are integrated in terms of activation and inhibition of genes. For the last decade, the study of such networks has been fueled by large-scale experiments and renewed attention from the theoretical field. Different models have been proposed to, for instance, investigate expression dynamics, explain the network topology we observe in bacteria and yeast, and for the analysis of evolvability and robustness of such networks. Yet how these gene regulatory networks evolve and become evolvable remains an open question.

An individual-oriented evolutionary model is used to shed light on this matter. Each individual has a genome from which its gene regulatory network is derived. Mutations, such as gene duplications and deletions, alter the genome, while the resulting network determines the gene expression pattern and hence fitness. With this protocol we let a population of individuals evolve under Darwinian selection in an environment that changes through time.

Our work demonstrates that long-term evolution of complex gene regulatory networks in a changing environment can lead to a striking increase in the efficiency of generating beneficial mutations. We show that the population evolves towards genotype-phenotype mappings that allow for an orchestrated network-wide change in the gene expression pattern, requiring only a few specific gene indels. The genes involved are hubs of the networks, or directly influencing the hubs. Moreover, throughout the evolutionary trajectory the networks maintain their mutational robustness. In other words, evolution in an alternating environment leads to a network that is sensitive to a small class of beneficial mutations, while the majority of mutations remain neutral: an example of evolution of evolvability.

## Author Summary

A cell receives signals both from its internal and external environment and responds by changing the expression of genes. In this manner the cell adjusts to heat, osmotic pressures and other circumstances during its lifetime. Over long timescales, the network of interacting genes and its regulatory actions also undergo evolutionary adaptation. Yet how do such networks evolve and become adapted?

In this paper we describe the study of a simple model of gene regulatory networks, focusing solely on evolutionary adaptation. We let a population of individuals evolve, while the external environment changes through time. To ensure evolution is the only source of adaptation, we do not provide the individuals with a sensor to the environment. We show that the interplay between the long-term process of evolution and short-term gene regulation dynamics leads to a striking increase in the efficiency of creating well-adapted offspring. Beneficial mutations become more frequent, nevertheless robustness to the majority of mutations is maintained. Thus we demonstrate a clear example of the evolution of evolvability.

## Introduction

Gene regulatory networks (GRNs) have become a successful tool for understanding the organization within cells and their dynamics. In GRNs information from the cell state and the outside environment is translated into a correctly timed expression of genes. As such, one may argue that GRNs are the nexus of physiological adaptations. However, as soon as the time scale of environmental change exceeds an individual's lifespan evolutionary adaptations will also play a role. In this work we concentrate exclusively on this evolutionary side of the equation.

GRNs have been studied extensively. Randomly generated networks have been investigated, for instance in deriving various characteristics of homogeneous random networks [1], assessing attractor landscapes [2] and evolutionary potential [2],[3],[4],[5],[6]. With the recent insights from the regulatory networks of model organisms, experimentally inspired networks have been investigated as well [7],[8],[9],[10],[11].

Mutational dynamics (i.e. neutral evolution) have been applied to GRNs in order to explain the global and local topology of GRNs [7],[12],[13],[14],[15]. Evolution with Darwinian selection and gene expression dynamics has been used to generate small biochemical networks realizing specific mathematical functions [16],[17] and to assess the requirements for evolving specific expression patterns [18]. Evolution has also been applied in the closely related areas of signal transduction pathways and metabolic regulation [19],[20],[21],[22],[23]. Predominantly these networks evolved to a fixed target. This has been successfully extended by evolving towards changing fitness regimes both in a genetic programming context [24] and for evolving electronic circuits [25]. The latter also demonstrated that alternating the evolutionary targets can decrease the total time needed to reach every target at least once [26].

In this work we alternate evolutionary targets and focus on the long-term evolution of adapting toward these targets. Reaching an evolutionary target is therefore only the first step: we study the effect of repeatedly evolving towards it. That is to say, we investigate the evolution of the genotype-phenotype mapping, from genome to network, on a longer time scale. To achieve this we do not directly operate on the network level. Instead we explicitly model a genome where mutations occur, and a network derived from this genome. We do not provide the individuals with direct input from the environment and consequently they are absolutely blind to environmental changes. Hence our observations are not influenced by physiological adaptations.

We concentrate our analysis on the evolution of evolvability. The concept of evolvability has been formalized in various ways [27],[28] and we define it as the efficiency of an organism in discovering beneficial mutants. Hence our question is whether evolution can modulate the mutational efficiency of ‘generating’ well-adapted offspring via the genotype-phenotype mapping. In other words, through the encoding of the network in the genome.

Evolutionary experiments with yeast, *S. cerevisiae*, resulted in compelling evidence for such evolvability [29]. Only a small number of mutations were needed to change the expression levels of many genes as well as causing an increase in fitness. In addition, while almost all strains showed gross chromosomal rearrangements, equivalent restructuring of the transcriptome and similar fitness gains were observed in strains with only minor mutations [30]. The observation of multiple, short mutational paths suggests the genetic system of yeast is capable of efficiently discovering advantageous adaptations. Similarly, in several independently evolved *E. coli* strains beneficial mutations on the same genes were found to influence large parts of the gene regulatory network [31]. These empirical studies strongly suggest the genotype-phenotype mapping itself is a product of evolution and may have become optimized to increase evolvability.

We show that in a changing environment individuals evolve their genotype-phenotype mapping such that they become more efficient at generating adaptive mutants. The evolvability manifests itself as a sensitivity to gene duplication and deletion mutations of one particular gene, an “evolutionary sensor”. The duplication or deletion of this gene results in the network switching its state toward the evolutionary target set by the environment. We show that these evolutionary sensor genes are either hubs of the regulatory network or directly provide input to a hub gene. In addition, our mapping from genome to network introduced a large degree of mutational neutrality. During the long-term evolutionary process the vast majority of mutations remained neutral, in other words, the population was constantly on a mutationally neutral network and evolvability hardly impacted the mutational robustness.

Summarizing, we show that in a dynamically changing environment long-term evolutionary processes and short-term gene regulation dynamics interact such that our gene regulatory networks become extremely efficient at generating advantageous mutations, while they remain mutationally robust.

## Results

**Figure 1:**
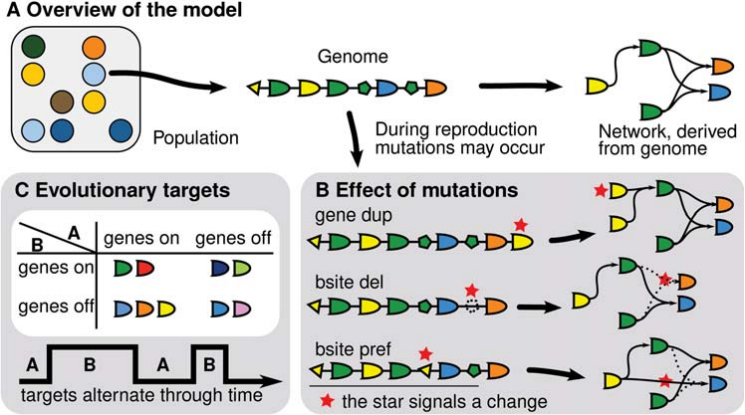
Overview of the model. (A) Simulations are run on a 150×50 lattice for 6·10^5^ time steps. The lattice harbors a population of genomes, where a genome is a linear chromosome of genes with binding sites. From a genome a Boolean threshold network is built. During each time step the network may update the expression level of the genes for 11 propagation steps. (B) The impact of several gene and binding site mutations is shown. The change in the genome and network topology is signaled by a red star. In a typical simulation the parameters are (per gene, binding site): gene duplication (dup) 2·10^−4^, deletion 3·10^−4^, threshold 5·10^−6^, binding site (bsite) duplication 2·10^−5^, innovation 1·10^−5^, deletion (del) 3·10^−5^, preference (pref) 2·10^−5^ and weight 2·10^−5^. See Model for an explanation on each type of mutation. (C) Typically the environment changes over time with a probability of *λ* = 3·10^−4^. The two evolutionary targets A and B determine which genes should be expressed (*on*) or inhibited (*off*). The result is four categories of genes; some should be always on, some should toggle their expression state and some should never be expressed. In a typical simulation, the target expression states are, from gene 0 to 19, A: 00011 11000 00000 11111 and B: 11010 01001 01100 01011.

To study the evolution of the genotype-phenotype mapping we employed an individual-oriented model (Figure 1). At the start it was initialized with a homogeneous population of genomes, from which gene regulatory networks were built (Figure 1A). On the genomes mutations occurred, such as gene and binding site duplications and deletions (indels), which influenced the network topologies of these individuals (Figure 1B). The individuals were selected for reproduction on the basis of their gene expression pattern, i.e. scoring if genes were correctly *on* or *off*.

As shown in Figure 1C, the environment determined the evolutionary target of the population. The goal was always to minimize the Hamming distance of the network state to a predefined expression state, yet which genes were to be turned *on* or *off* changed through time. For the simulations we selected by hand two network attractors from the initial network as the evolutionary targets. In other words, the population adapted to an attractor state and the environment alternated the attractor over time. As mentioned in the Introduction, the individuals could not sense these changes in the environment.

Due to the computationally intensive nature of our model the main results presented are based on a set of 15 replicate runs. In all cases the population evolved evolvability, and 11 (73%) runs showed so-called evolutionary sensors (ES). The latter ones are our focus in this work. For an in-depth analysis we randomly selected a single run with an evolutionary sensor, which we refer to as a typical run. In the four runs in which the population failed to reach the solution of an evolutionary sensor, we have been unable to pinpoint a specific strategy.

### Evolving to the Targets

**Figure 2:**
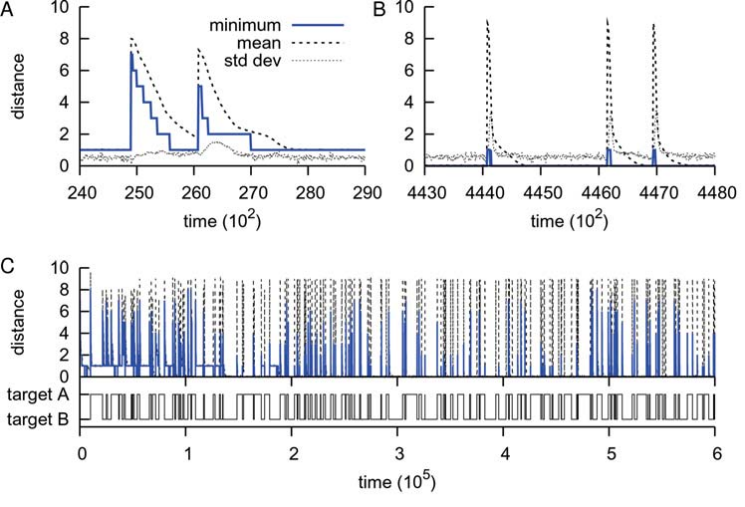
A typical run. (A,B) Close-up of the population dynamics. The population is minimizing the Hamming distance to the evolutionary target. For two intervals (one at the start of the run, the other to the end), the minimum and mean population distance with the standard deviation are plotted at a resolution of 10 time steps. (C) An overview of the entire run. The top panel shows the population minimum and mean distances as in figure A and B, while the bottom panel shows the random timing of alternations between the evolutionary targets. There were 191 switches between the two targets.

During a simulation the population repeatedly adapted as the environment changed. The individuals in the initial population had the desired network states as attractors in their attractor space, yet we observed that in the beginning they were unable to reach these gene expression patterns (Figure 2A, the population reached distance 1). That is to say at the start adaptation was slow and unsuccessful, though eventually the population evolved a swift mode of switching correctly between attractors. As can be observed in Figure 2B, when the environment switched, the population had to change the expression state of nine genes, which caused the mean Hamming distance to jump to 9, while within the resolution of 10 time steps the minimum already jumped back to 1. Hence the best individuals had virtually immediately activated and/or inhibited eight genes via a mutational adaptation: a clear sign of the evolution of evolvability.

**Figure 3:**
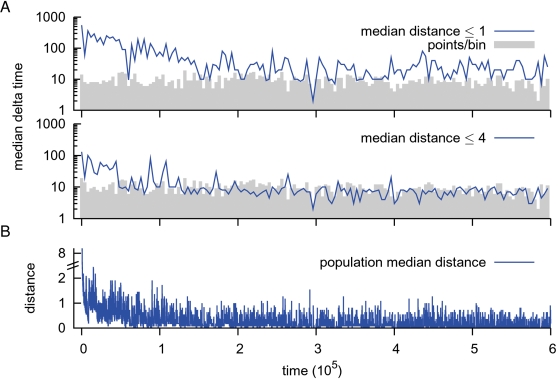
Evolving to the targets. (A) The median time differences (delta time) of the 11 runs are plotted. After an environmental change we recorded the time to reach at least the mentioned Hamming distance (1 and 4) from a previous higher one at a resolution of 10 time steps. The top panel shows the median time to almost reach the evolutionary target, while the bottom panel shows the median time to get at least halfway between the two evolutionary targets. Due to the random timing of environmental changes in the runs, we binned the time differences and show the number of points per bin in the background of both figures (bin size = 4·10^3^ time steps). Note the logarithmic scale of the ordinate. (B) The median of the population median Hamming distances for the 11 runs. Due to the random environmental changes of all the different runs, the populations appear not to reach the evolutionary targets. However, as shown in Figure 2 in a single run it is clearly visible that the populations do so.

To assess the improvement we calculated for all runs the time differences between consecutive Hamming distances to the evolutionary target (Figure 3A). Sustained gains in the speed of adaptation were observed until *t*≈1.2·10^5^ for reaching at least a distance ≤4, and until *t*≈2·10^5^ for a distance ≤1. Additionally, as a signature of the global evolutionary dynamics, we have taken the median of the population median distances (Figure 3B). In agreement with the time differences shown in Figure 3A, until *t*≈2·10^5^ the populations improved their ability of simply reaching the evolutionary targets, followed by a long transient of slowly decreasing population median distances.

Concluding, after an initial phase, the population had evolved a ten-fold improvement in its mutational speed of alternating network states. In other words, the individuals had arrived at a genotype-phenotype mapping that allowed for rapid and accurate switching. We now turn our focus to the long-term evolutionary dynamics.

### An Evolutionary Sensor

**Figure 4:**
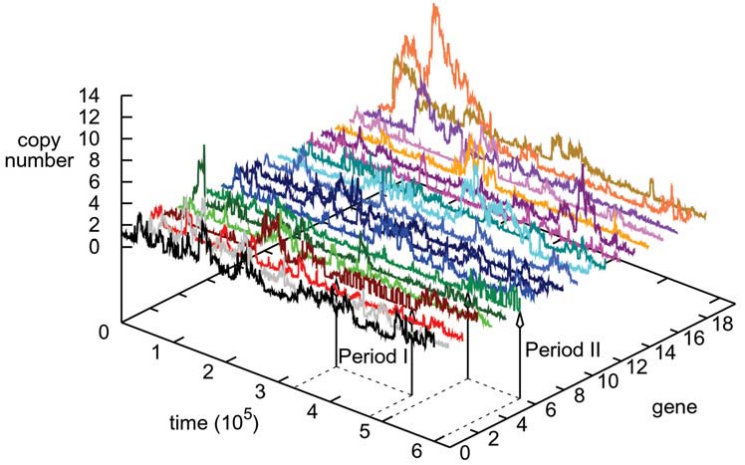
The change in copy number for each gene. For each of the 20 different gene types the average copy number in the population is plotted through time (see Model for an elaboration on the concept of gene types). Two intervals are highlighted: *Period I* stretches from *t* = 3.4·10^5^ to 4.7·10^5^, where gene 3 shows switching behavior and *Period II* from *t* = 5·10^5^ to 6·10^5^ where gene 6 takes over as evolutionary sensor. See also Figure S1 and Figure S2 for other runs with ESs.

As a proxy for the genome content of individuals we measured the average copy number of genes in the population (Figure 4). Besides the drift of gene 18, two remarkable periods were observed in this run. In *Period I* the copy number of gene 3 alternated between 1 and 2 as the environment switched 44 times back and forth between the two attractors. The behavior was lost around *t* = 4.7·10^5^, and gene 6 quickly took over the same behavior of switching copy numbers. The remaining part of the simulation was marked by *Period II* and contained 38 alternations of the evolutionary target. Immediately the hypothesis arose that these genes were responsible for the multiple events of extremely rapid evolutionary adaptation.

In order to verify the validity of the hypothesis a detailed picture of the evolutionary dynamics was needed. Therefore we closely examined the evolutionary process by performing an ancestor trace (see Model). Due to adaptive mutants sweeping the population after each environmental change, the entire population had a recent single common ancestor, and hence by looking at a single ancestor trace, we essentially looked at the one lineage that has survived from the start. This complementary analysis allowed us to characterize genes 3 and 6 in much more detail: What their impact was on the adaptation, how networks utilized them to switch the gene expression state and how they altered the local mutational landscape.

#### Adapting with a evolutionary sensor

**Figure 5:**
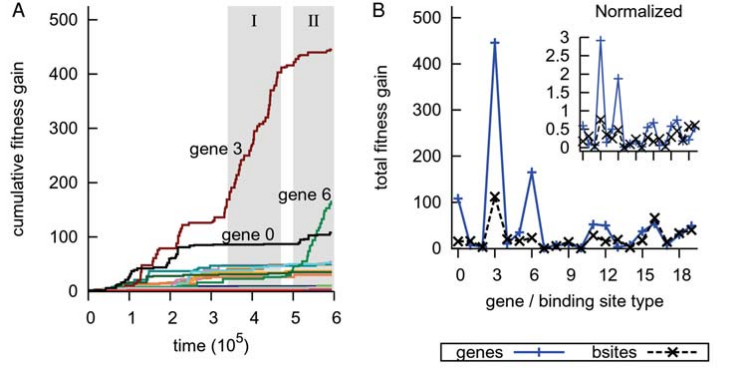
Fitness gained per gene or binding site type. From an ancestor trace the adaptive mutations were categorized by gene or binding site identification tag. (A) The cumulative fitness gain of genes is shown through time. Highlighted are *Period I* and *Period II*. (B) The total fitness gain of gene and binding site mutations is shown. The *inset* figure shows the contributions normalized by the number of mutations of each type. Note that the peak at binding site 3 is a fluke; during the transition from *Period I* to *Period II* binding sites with a preference for gene 3 were involved in a few very effective mutations (data not shown).

Figure 5 shows the cumulative fitness gain over time (A) and final fitness gain (B) of mutational events. We could see that the involvement of genes 3 and 6 in adapting to the environment was unmistakable. Initially the networks in the ancestor trace appeared to avoid gene mutations and from *t*≈1·10^5^ to 3.4·10^5^ a variety of genes was used. Clearly in both *Period I* and *Period II* the evolutionary sensor genes accounted for the large majority of adaptive mutations (Figure 5A).

In Figure 5B we see that a few genes played pivotal roles in this run, while binding site mutations had only a minor effect on the dynamics. The inset of Figure 5B shows that both gene 3 and 6 also had a high effect per mutation. Interestingly, from Figure 5A we found gene 3 to have been a rather ‘dominant’ gene throughout the run, while gene 6 was active solely during the last part. This implies that before gene 6 became a sensor its mutations were mostly deleterious and hence were hardly encountered in the ancestor trace.

With respect to our initial hypothesis: indeed gene 3 and 6 played a central role in the long-term evolutionary adaptation through their copy number alterations and accompanying fitness gains. However, the observations in the above paragraph indicate that the question why specifically these genes became sensors is not trivial.

#### A case study at time = 457755

**Figure 6:**
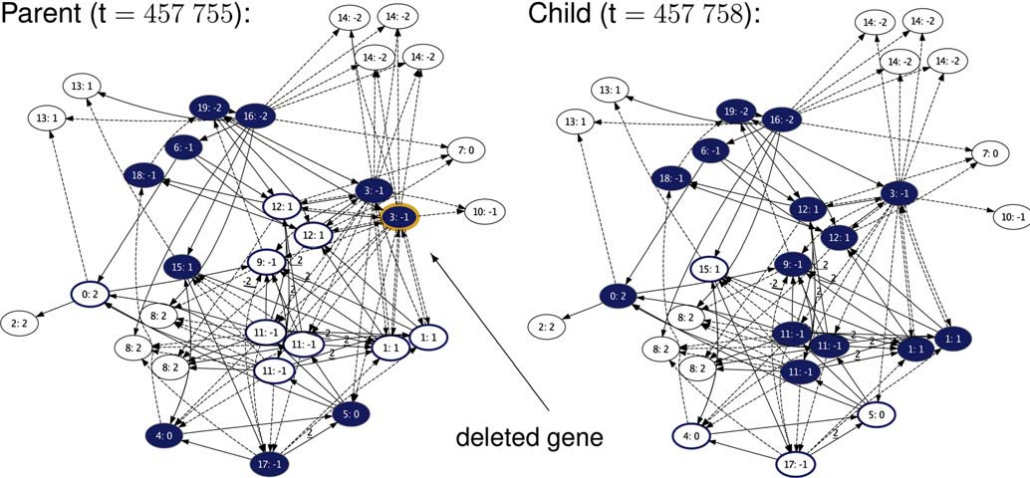
A network switching attractor. Genes are represented by nodes, labeled as (identification tag: expression threshold), colored blue if active, or a blue outline if they are active in the opposite attractor. Activating interactions are solid edges, inhibiting ones are dashed. The deletion of gene 3 silences genes 4, 5, 15 and 17 and activates 0, 1, 9, 11 and 12, which is exactly a switch between the two evolutionary targets. As we study a long-term process, the networks are representative of the ‘final solution’ that the population evolved to (a switch guided by gene 6 is shown in Figure S4). For visibility, both networks were pruned for interactions originating from genes that are always silent and for parallel interactions that cancel out.

As an example of how a single mutation pushed a network from one evolutionary target to the other, we selected two consecutive individuals from the ancestor trace. As shown in Figure 6 they differed by a deletion of gene 3.

Both genes 3 had been active in the parent network, thus the deletion caused a reduced sum of inputs at the target genes 1, 5, 9, 11, 12, 14. Of the 31 genes 13 were targets of gene 3, yet most had compensatory input from other genes and as a result only genes of type 11 were affected. As gene 5 was still active, they were no longer sufficiently repressed and therefore the dosage effect of lacking one gene 3 activated 11.

It followed that genes 11 activated gene 0 and turned off gene 4, 15 and 17, which in turn silenced gene 5. Furthermore, once gene 17 was off, 11 turned on 1 and 9. Interestingly the two copies of gene 12 were activated via different pathways. One is brought to expression via genes 11, the other via genes 1. Thus within four propagation steps of the network, the individual changed the expression of nine genes and ended in a different attractor.

### The Local Mutational Landscape

We have shown that evolvability can evolve in a changing environment and by what kind of mutations this process takes place. Now we study the changes in the local mutational landscape of the individuals as a direct measure of the evolved genotype-phenotype mapping.

All one-point mutants were generated for each individual in the ancestor lineage and, after allowing the mutant networks to settle in a new (stable) gene expression state, we computed the Hamming distance of the mutant network to the opposite evolutionary target. Next, we subtracted the mutant's distance from the individual's distance to the opposite target, which gave us the improvement of the mutant in Hamming-distance units. By opposite target is meant the network state that the individual did *not* evolve to at the time of birth. Since the population was for a large part of the simulation close to either target, we looked for signs of evolvability in the local mutational neighborhood of these individuals; how well they were able to switch to the opposite target.

**Figure 7:**
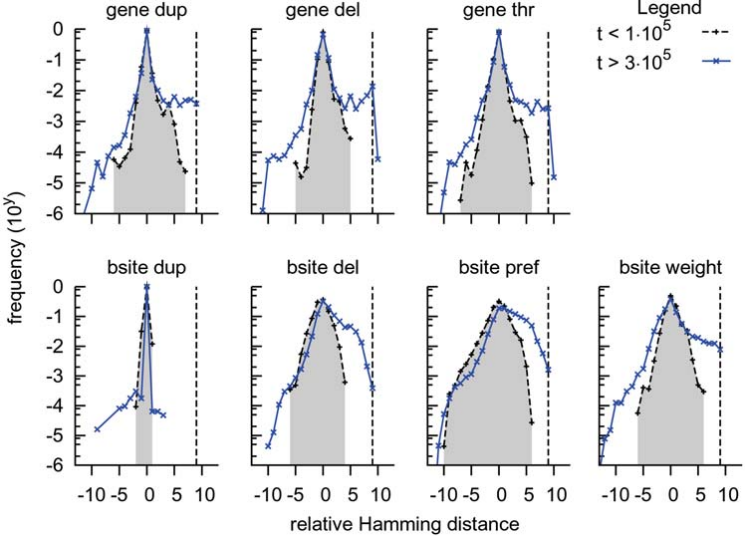
Hamming distance improvement to the opposite evolutionary target. Each sub-figure gives for a mutational operator the frequency plot of the Hamming distance of a mutant compared to its ancestor with respect to the opposite evolutionary target. Positive distances signal that mutants are closer to the target, a distance of 0 is a neutral mutant and negative distances indicate mutants are farther from the target. The evolutionary targets have a distance 9 from each other, indicated by the vertical dotted line. The black dotted line shows a frequency plot integrated over all ancestor trace individuals until *t* = 1·10^5^, the blue solid line integrates from *t* = 3·10^5^ to 6·10^5^. Note that the ordinate is in log-scale and that binding site innovations have been grouped with duplications. (*dup* duplication, *del* deletion, *thr* threshold, *bsite* binding site) and *pref* binding preference.

In Figure 7 we observe that for every type of mutation the mutants initially (*t*<1·10^5^) peaked at distance 0 from the ancestor and they were approximately symmetrically distributed around this peak. Thus the vast majority of the one-point mutations was neutral, and few mutations allowed the individuals to change their gene expression either towards or from the opposite target. In the second half of the simulation (*t*>3·10^5^), where the sensors dominated the evolutionary adaptation, the distribution of mutants was strikingly different. Still the majority of mutant networks ended in the ancestor's state, indicating a maintained mutational robustness against gene expression changes. But in contrast to the initial variety of mutants, the ones that were adaptive for the opposite target were now overrepresented. Except for binding site duplications, all types of mutations showed a difference of several magnitudes for mutating towards the opposite target. Especially the gene mutations (duplication, deletion and threshold changes) and binding site weight changes were capable of generating adaptive offspring close to the opposite target. The evolutionary change in the effect of binding site deletions and binding preference was less focused. They became less likely to mutate away from the environmental target and more likely to mutate (a bit) towards the target. In other words they influenced only a few genes, but with a high probability of improving in the direction of the target. These observations suggest that genes performed the large mutations, while binding site mutations resulted mainly in small adaptations (which is nicely in concordance with Figure 5B).

Thus it appears that the evolution of the genotype-phenotype mapping maintained mutational neutrality that is inherently present in the mapping, while it increased the number of one-point mutants near the opposite evolutionary target.

### Mutations and Neutrality

The first trial simulations we ran on a grid of 100×50. These rather small populations reached both evolutionary targets and showed a tendency to develop evolvability, but were never able to keep it for more than a few environmental switches. Neutral mutations were accumulating in the population (data not shown), and the (secondary) evolutionary process of creating an ES was faced with its own Muller's ratchet.

**Figure 8:**
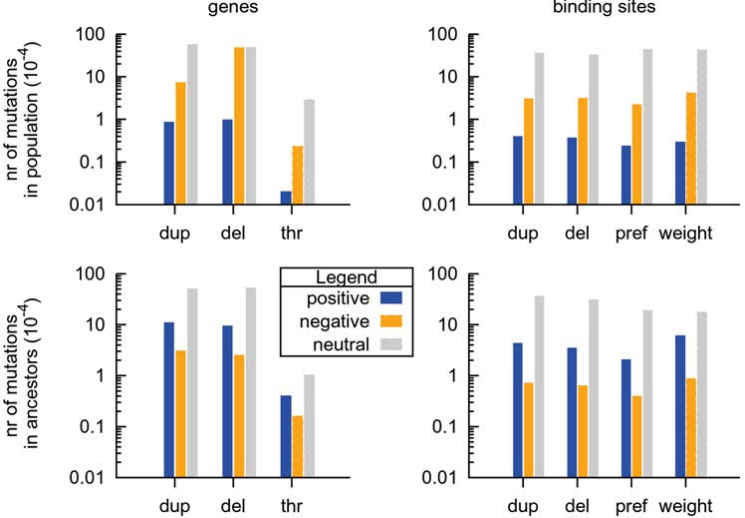
Mutations categorized by their immediate fitness effect. The mutations are: duplication (dup), deletion (del), threshold (thr), binding preference (pref) and weight. Per type three categories are distinguished: positive effect (gaining fitness), negative effect and neutral. Each bar is an average over the entire run per individual in the population (top panels) and per individual from the ancestor trace (bottom panels). In the population 4.10·10^9^ individuals were born and of them 1.26·10^5^ belonged to the ancestor lineage. The total number of events is comparable for genes and binding sites, in the ancestor it is respectively 1660 and 1545. Note that the ordinate is in log-scale, that binding site innovations have been grouped with duplications and that the large number of deleterious gene deletions in the population (compared to the binding sites as well as the ancestor's gene mutations) is explained by the lethality of missing a gene type.

Subsequently we enlarged the lattice, which lead to the presented results. We recorded the mutations both on the level of the population and the ancestor trace and categorized them by the direct fitness effect. Naturally, the individuals in the ancestor trace (in short ‘ancestors’) received a magnitude more beneficial mutations than the average individual in the population as shown in Figure 8. Nevertheless the ancestors also appeared to have had rather many deleterious mutations. The majority of these mutations had their effect altered during the lifetime of an individual: an environmental switch of the evolutionary target turned the mutation into a beneficial one. We found that 70/106 deleterious mutations were in fact advantageous.

**Figure 9:**
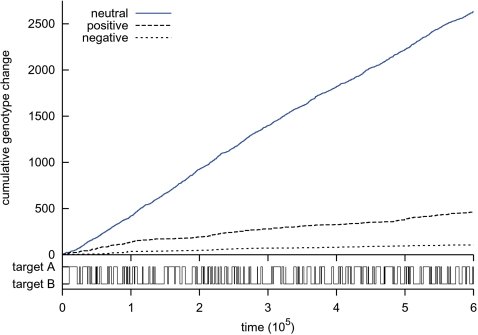
Traveling on the neutral network. The top panel shows the accumulated genotype changes categorized by their effect on fitness (positive, negative and neutral). The bottom panel is a reminder of the switching of the environment between the two targets.

Most interestingly the majority of mutations was still neutral, as we also observed in Figure 7. By comparing in Figure 8 the top panels with the bottom ones, we observed that both the ancestors and the average individual from the population had fixed a similar number of neutral mutations in their genomes. As shown in Figure 9 there was a constant rate, almost clock-like, of acceptance of neutral mutations. From this we may draw two conclusions: (a) the population was drifting on a neutral network of network topologies [32],[4],[5] and (b) even though the networks achieved greater evolvability, the neutrality of their mutational neighborhood was largely maintained.

### Properties of an Evolutionary Sensor

One way to rephrase the evolution of the genotype-phenotype mapping is to say it is the evolution of the network topology. The identification of the ESs was a dynamic characterization of how the network was shaped and altered. Thus it is interesting to study the properties of these sensor genes with respect to the network topology. In order to provide a more general and cohesive picture, we characterized several properties of the ESs in all the 11 runs in which these evolved.

First of all, in the typical run gene 3 and 6 needed to be expressed in both environmental targets, which enabled the networks to create a dosage effect that changed gene expressions. As shown in the list of runs in Figure 6E such constitutively expressed genes (genes 3, 6, 16, 18 and 19) are clearly overrepresented among the sensor genes. So genes that should be always *on* were favored by evolution.

Next, if we consider that the evolutionary process must structure the raw material of binding sites and genes in order to produce the correct expression state of the genes, the layer in-between the genotype and phenotype, i.e. the network topology, should provide key insights into the evolvability we observe. Therefore we related two important characteristics of nodes in a network, the outdegree and indegree, to the already known ‘dynamic’ property of the gene copy number change. The latter we looked into indirectly in the section “Adapting with an Evolutionary Sensor”, where we found a positive association of ESs with gene copy number change. That is to say, the more a gene alters its copy number, the more likely it is to be an evolutionary sensor.

First of all, for each run we visually identified ESs by their copy number changes in graphs like Figure 6. Then we selected from the simulations two intervals. For the long term evolutionary dynamics we took the second half of the runs (*t*>3·10^5^), and as a reference we picked the initial period until *t* = 1·10^5^. For a gene to influence the state of the network, it needs an outdegree. Thus we first studied the change in outdegree of the ESs. As shown in Figure 6A initially their copy number change and outdegree were uniformly distributed and one could not distinguish ESs from other genes. This strongly contrasts to Figure 6B, where the majority of ESs had evolved to a high copy number change and large outdegree. We still observed four ESs with a combined low copy number change and outdegree. This is explained by the fact that their ES behavior was observed only for a short period of time. For instance, gene 6, which we discussed previously, is among these genes. The secondary evolutionary process of creating ESs had been acting for too short a time to distinguish these genes from the rest.

**Figure 10:**
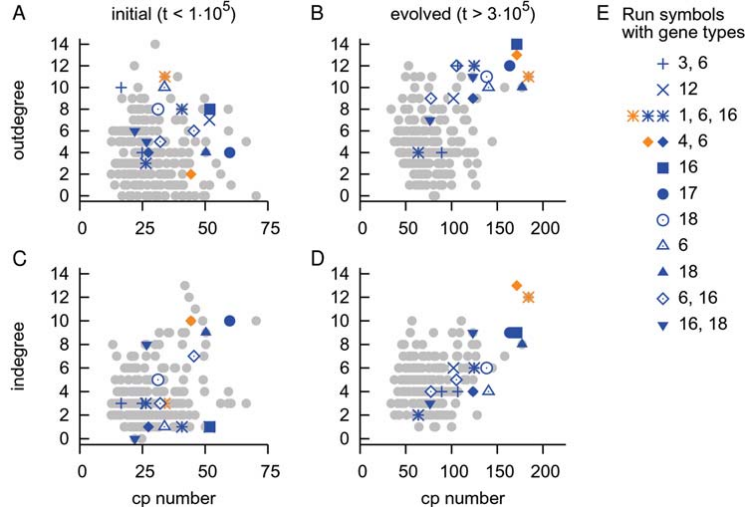
Scatter plots of gene properties. All data points are population averages per gene. See Model for details. Plotted are the initial (A,C) and evolved distributions (B,D) of accumulated copy number change against outdegrees (A,B) and indegrees (C,D) of each gene. The accumulated copy number change (cp number) is a measure for how often a gene is duplicated or deleted in the entire population, thus showing fixation of such mutations in the population (i.e. indicating it may have been adaptive). The outdegree and indegree are topological properties of genes in a gene regulatory network indicating respectively how many genes they influence and by how many they are influenced. In each subfigure genes that we identified as ESs are shown in *blue*, while to indicate hub genes receiving input from ESs *orange* is used. E. A list of the runs. For each run a different symbol is used, with the gene types of the involved sensors/hubs. Constitutively expressed genes are 3, 6, 16, 18, 19.

Two runs showed a different strategy. By sampling networks through time in the third run (third from the list in Figure 10E) we established that gene 6 and 16 had been providing input to gene 1, which is a hub gene. The network state change involved either copying an ES or a state-switching hub gene. Thus the network was controlled by two hub genes, one of which was an ES. A similar scenario holds for the other case (fourth run from top in Figure 6E) (data not shown).

Secondly we studied the indegree of genes (Figure 10C and D). In the beginning copy number and indegree were uniformly distributed, as was the case for the outdegree. However, unlike the outdegree, we did not observe any clear long-term evolutionary effects on the indegree. In other words, there was only a selective increase in outdegree. Moreover the fact that such a signature of the network topology is still visible after averaging over populations and over the chosen time intervals is astonishing and shows that the result is robust.

Of the 15 runs, we already mentioned four did not show any signature of ESs. These runs also had no genes which evolved towards high outdegree (data not shown). Thus the topological characterization provides a general procedure for discriminating among runs with and without ESs, and for identifying genes as evolutionary sensors.

### A General Strategy

We expanded the scope of the problem by introducing a third evolutionary target. Instead of a “simple” toggling between two attractors, evolution needed to generalize the process of duplicating and deleting genes in order to change the network state. Although observing a clear duplication and deletion pattern for an evolutionary sensor as in Figure 4 was hard, the scatter plot of outdegree and indegree against copy number change showed evolutionary sensors (Figure S3). That is to say, the genes most likely to be an evolutionary sensor, i.e. genes which should always be expressed, showed ES behavior with respect to outdegree and indegree. From this we conclude that an ‘evolutionary sensor’ strategy has been applied in this extended case as well.

Finally, the results that we have presented in this work were cross-checked against a variety of 8 initial networks and different evolutionary targets with qualitatively equivalent outcomes. The ancestor trace analysis was checked against ancestor traces of two other randomly selected runs with a sensor, again with qualitatively the same results. Additionally, a broad range of mutation rates and environmental change rates resulted in networks with evolutionary sensors (Text S1).

## Discussion

The accepted framework in which evolution operates is that mutations are random events and selection acts on the generated variation. Nonetheless, even if we assume mutations are random, their phenotypic effect may be strongly biased. In our simple model of GRN evolution we recognized that only a specific subset of mutations was selected for. The networks had become sensitive to the indel mutations of a particular gene, the evolutionary sensor. That is to say, the genotype-phenotype mapping from genome to network had evolved such that a small class of mutations was adaptive and therefore repeatedly observed. This demonstrates a clear example of mutational priming and hence of evolution of evolvability [33].

Previously we have studied a similar process at the level of the genome, where a genome composed of genes and transposons structured itself in a manner that favored mutations at specific locations on the chromosome [34]. In our current model we lack the transposons and consequently mutations are not biased to a location on the genome. Instead the networks have been shaped by evolution to allow for swift adaptation to different environments. Over time the evolutionary sensor genes became hubs of the regulatory networks. In contrast to the genome being shaped by evolution, there has been a structuring of the evolutionary substrate on a higher hierarchical level, the network topology.

### Attractor Landscapes

**Figure 11:**
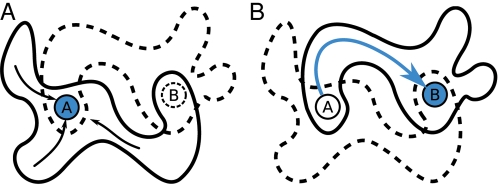
The local attractor landscape around target *A* and *B*. (A) The network is in attractor *A*, and its basin of attraction is shown by the black arrows and the solid-outlined ‘cloud’ around them. (B) Attractor *B* has come into existence, forcing the network state of attractor *A* to propagate through the basin of attraction into *B*, as shown by the blue arrow.

The case of evolvability that we presented is elegantly explained in terms of the attractor landscape and its basins of attraction. A conceptual representation is shown in Figure 11, where in the left panel the network is in target attractor A. Attractor B need not exist at this moment, but due to a gene duplication or deletion of the evolutionary sensor, it will be created in tandem with the destruction of attractor A. In this manner the network state is suddenly in the basin of attraction of target B and the network ends in the correct state, target B.

A priori we did not anticipate such a dynamic attractor landscape. Recent work on the evolvability of networks had focused mainly on conserving old attractors, while gaining new ones [2], or keeping gene expression patterns, while altering the interactions [4],[5]. Our work complements these, as we show networks are also capable of (re)generating ‘known’ attractors that are not necessarily present in the current attractor landscape. Remarkably, the networks have evolved toward a topology that allows them to establish and move to a new attractor and to do so in a reversible fashion.

### Discussing the Model

With respect to our modeling formalism we would like to highlight two assumptions. First of all, we let binding sites determine via their weight whether the effect of a transcription factor (TF) is activating or inhibiting. One could argue this should be a shared decision or perhaps rather that binding sites should be passive, as TFs are generally regarded as being either only activating or inhibitory and not a mixture of the two. On the other hand the yeast cell cycle regulation does show that TFs can have opposite effects on different genes [9]. Because we only model the TFs of a gene regulatory network, it is not unrealistic to allow for a more delicate tuning among them.

As mentioned above, the genomes and networks constitute the transcriptional core of a complete gene regulatory network. Hence an evolutionary target defines which combination of transcription factors is required to activate the correct (but not explicitly present) target genes. A few exploratory simulations with explicit target genes suggest the more ‘realistic’ case creates an easier task for evolution. One plausible explanation is that not predefining the exact wiring of which TF controls which target genes provides extra flexibility to the mutation-selection process. In that case, we have presented here a worse-case scenario of GRN evolution.

### Generalizing the Model

In gene expression dynamics noise is an important player (especially if one looks into the details). It is known that evolution may even exploit these random fluctuations, for instance to switch between distinct gene expression patterns [35]. We explored how evolvability was affected by noisy gene expression in our model. With a certain probability a gene toggled its state during the lifetime of an individual. It is important to realize that completely activating or silencing a gene is a strong type of noise, however evolution of evolvability was still observed. In three runs with high amounts of noise (*p* = 0.04, which translates to more than one gene affected per network propagation step) we identified evolvability in all cases and an evolutionary sensor in one of them. Thus the model appears to be able to cope with expression noise.

Secondly, instead of copying the expression state of a gene when it is duplicated, we initially silenced the new copy both as a biologically more sound setting and to test the resilience of the evolutionary process in discovering the evolutionary sensor. The latter relates to the observation that copying a gene basically results in a dosage effect of the sensor gene that then percolates the network. We still observed evolution of evolvability and the accompanying sensors. The straightforward solution was to have a negative gene expression threshold for the evolutionary sensor. As long as the sensor gene received more activating than inhibitory inputs, the copy would start expressing with a delay of one time step compared to the original setting.

The last alteration we performed involved the copying of the gene expression pattern at reproduction. If one imagines a cell splitting into two daughter cells, it sounds reasonable to copy the state of the genes. However we ignored the cell cycle and therefore we introduced a birth state for each gene that mimics the starting point of the cell cycle. Again we found evolvability and the presence of a sensor gene.

### From In Silico to In Vivo

In order to focus on evolution, we have imposed the restriction of no environmental sensor. This implies that the gene expression state cannot be pushed out of an attractor; if the network has to switch its state, the only option is to turn the current attractor into a transient state. Naturally, the question arises whether organisms actually perform gene expression changes via mutations. In bacteria it is known that specialized DNA recombination events, such as DNA inversions [36], replication slippage in combination with methylation patterning [37] and other rearrangement events [38] underlie phenotypic switching. Most importantly, these occur without prior environmental signals. This allows for a heterogeneous population that is resistant to sudden environmental changes [39]. Whether such mutational mechanisms were involved in the evolutionary adaptation of the *S. cerevisiae* strains from which we have drawn our inspiration remains an open question. Still the more general idea of using, in a reversible manner, mutations to alter gene expression and consequently the phenotype seems to be a widespread mechanism.

### Summary

By combining the time scales of evolution and gene expression with a dynamic environment, we have shown that networks become evolvable while their robustness to mutations is maintained. Mutational mechanisms to stochastically switch phenotype are applied by bacteria and single-celled eukaryotes, and we have demonstrated a scenario for the evolution of such survival mechanisms. In addition, our work provides a new search-image with respect to the effect of mutations on short-term evolutionary adaptation, which may be of importance in an upcoming field like synthetic biology.

## Model

We study an individual-oriented model with a population on a lattice subjected to an environment that changes over time (see Figure 1). The simulation is initialized with a homogeneous population and usually run for 6·10^5^ time steps, during which the environment alternates between two evolutionary targets, that is two gene expression patterns, according to a Poisson process (usually *λ* = 3·10^−4^).

A lattice has been used for two reasons. Firstly, it enables a computationally efficient method for competition among individuals and, secondly, it is biologically sound, as organisms virtually always live in a spatial system with a certain degree of locality. Given a default lattice size of 150×50 and a fixed death rate for each individual of 0.1, the population size averages around 6750.

### Individual with a Genome

Each individual starts with a linear chromosome containing *n* different genes (*n* = 20) and on average 2 binding sites per gene. The network is derived from this chromosome with genes as nodes and interactions between genes defined by which gene binds to which binding site. The fitness of an individual is defined on the level of network states, i.e. which genes are activated or inhibited. Reproduction of an individual is based on this fitness. Note that we only model the transcription factors explicitly, and hence assume a certain combination of activated transcription factors would result in the correct activation and inhibition of ‘phantom’ target genes.

### Network

At the start of the simulation all individuals have the same network. The network has been selected from a pool of randomly generated networks according to the following criteria: (1) the network is connected, (2) there are no parallel edges in the network, (3) the average Hamming distance between the attractors with a basin size >10 is ≥5. The evolutionary targets have been chosen at random from the available attractors in the network, with a Hamming distance between them >6.

#### Genes and binding sites

The network consists of genes with interactions among them. A gene has a state of expression *s* (on = 1, off = 0), a threshold *θ* ∈{−2,−1,0,1,2} and an identification tag *t* ∈{0,1,2,…,*n*}. Binding sites specify which gene may bind to them via their own identification tag (i.e. if tags are equal), which is called the binding preference. They also determine the type of interaction *w*: *activation* (*w* = 1) or *inhibition* (*w* = −1). If there are multiple copies of a binding site present in the upstream region of a gene, there will be parallel edges in the resulting network. Symmetrically, if there are multiple copies of a gene, they all bind to a binding site.

#### Gene types

The duplicates of a gene all have the same identification tag. As mentioned above they behave equivalently in terms of binding and, as described below, we map the expression states of all genes with the same tag to one state. Therefore we introduce the concept of a gene type: a group of genes which all have the same identification tag.

We do not allow for new types, nor do we allow genes to change their identification tag. It creates a closed system of gene types that simplifies the definition of the evolutionary targets, i.e. the network states the population has to evolve to. Hence the copies of a gene may be viewed as constituting a family of transcription factors.

Throughout the text we use both gene and gene type for the collection of genes with the same identification tag, unless this would result in ambiguities. In similar fashion we group binding sites by their identification tag.

#### Updating the network

On the network the gene expression dynamics are defined. Similar to classical Boolean networks, the genes in the network are updated in parallel. However, as it is a threshold network, for a gene *i* its state of expression *s_i_* at time *t*+1 is defined as:
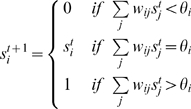
This network approach has been successfully applied to the yeast cell-cycle network [9].

### Fitness and Reproduction

The fitness *f* of an individual is based on the Hamming distance *D* between the current state of its genes and the target network state as defined by the environment. If gene indel mutations have resulted in multiple copies of a gene (all of them have the same tag), that type of gene is regarded as *on* if at least one of the copies is on, and *off* if all copies are off. This is based on the fact that duplicated genes are usually capable of substituting for each other. Both missing a gene and not having any gene in the network activated are lethal. The Hamming distance is normalized and rewritten as a similarity measure. In formula the fitness is defined as:
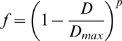



Selection pressure is increased by raising *f* to a power *p*, which increases the chance that a beneficial mutant spreads in the population. We fix *p* = 10, which closely resembles an exponential function in the range [0,1].

The fitness determines the probability of producing offspring *r*, if there is an empty grid cell in the neighborhood to place the offspring. Given such an empty location, the eight neighboring individuals, called *nbh*, compete on basis of their fitness score *f*

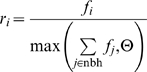



The threshold Θ (fixed at 0.4*^p^*) creates the probability that if there are only a few individuals in *nbh* or these individuals are very unfit, nothing may happen. Given the relative fitness *r_i_* of each individual in the neighborhood *nbh*, one is selected according to the fitness proportional selection scheme. Reproduction itself encompasses copying the chromosome, mutating and dividing into two daughters. The state of the genes is copied as well, in other words there is inheritance of the network state. Subsequently one of the two daughters replaces the parent, the other is placed in the empty grid cell.

### Mutational Events

While selection acts on the network, mutations act on the chromosomes. During reproduction the genome is duplicated, creating a diploid individual, after which mutations may occur on both chromosomes. We have defined the following events on genes:

- *duplication*: a gene with its binding sites is copied to a random location on one of the chromosomes. The expression state of the gene is copied as well,
- *deletion*: a gene with its binding sites is removed from the chromosome,
- *threshold change*: the current gene expression threshold is changed to a randomly chosen, valid, other value,

Binding sites have several types of mutations as well:

- *duplication*: a binding site is copied to the upstream region of a random gene in the genome. This introduces a new connection in the network,
- *deletion*: a binding site is deleted. If one or more genes bind to the deleted binding site, multiple connections are deleted in the resulting network,
- *innovation*: a new binding site is inserted in the upstream region of a random gene, with a random weight and a random binding preference,
- *weight change*: a binding site toggles from being activating to inhibiting or vice versa,
- *preference change*: a binding site changes its binding preference, in other words the gene type that binds to the binding site is changed. This may involve multiple connections being deleted and created in the network.

In our simulations we assume deletions should occur more often than duplications for two reasons. Firstly, the growth of a genome is bounded in this manner and, secondly, deleting a gene or binding site is regarded as an inherently ‘easier’ task than duplicating it.

### Ancestor Tracing

We trace lineages of individuals by attaching to them a unique identification and recording the parent-child relationships. The result is a “perfect fossil record”. A single trace from one of the fittest individuals in the final population back to the initial population allows us to dissect the exact mutational dynamics, to calculate attractor state spaces of the networks and see their evolution. It also enables us to perform mutational experiments on each individual and to visualize the resulting mutational landscapes. In order to perform such ancestor tracings we only consider asexual reproduction in our model.

### Analyzing Indegree and Outdegree

During a simulation, population averages of the gene copy number, indegree and outdegree are saved to disk categorized by gene type. At a resolution of 1000 time steps we get a good view of the general evolutionary trends.

As a measure for the amount of change in gene copy number, we sum, for each gene type, the absolute differences between adjacent sampling points. The more a gene fluctuated in copy number, the higher the sum. Hence evolutionary sensors have a tendency for high sums, as do genes that drift a lot. Both indegree and outdegree averages were binned in histograms, and from the resulting distributions the medians were taken as a representative number.

By using the above described procedure for the interval [0, 1·10^5^) and [3·10^5^, 6·10^5^) we constructed Figure 6. Evolutionary sensors were identified by hand using graphs as Figure 6 and then marked in the graphs of copy number against indegree and outdegree.

## Supporting Information

Text S1 Parameter Dependencies, Mutation Rates, and Environmental Rate of Change
(0.02 MB PDF)

Figure S1 For each of the 20 different types, the average copy number in the population is plotted through time. There are two clear ESs visible in this run. From *t ≈* 0.5 *·* 10^5^ to 2.5 *·* 10^5^ gene 16 is the ES, and from *t*  =  3 *·* 10^5^ to 6 *·* 10^5^ gene 6 is the sensor. With the exception of gene 1, most of the other genes do not show large fluctuations in the long term. This run is number ten in Figure 10E (first before last).
(0.19 MB PDF)

Figure S2 For each of the 20 different types, the average copy number in the population is plotted through time. In contrast to Figure S1, we observe more fluctuations in copy numbers. Gene 18 is the ES, and for most of the run its copy number alternates between 1 and 3, or 2 and 4, which results in a fuzzier signal. Still, the gene is responsible for the adaptation. After *t* ≈ 5 · 10^5^, gene 18 shows the clear-cut behavior of an ES as we have seen in Figure 4 and Figure S1. This run is number nine in Figure 10E (second before last).
(0.20 MB PDF)

Figure S3 Scatter Plots of Gene Properties for the Case of Three Evolutionary Targets. All data points are population averages per gene type. See Model for details. (A–D) Plotted are the initial (A,C) and evolved distributions (B,D) of accumulated copy number change against outdegrees (A,B) and indegrees (C,D) of each gene. The accumulated copy number change (cp number) is a measure for how often a gene is duplicated or deleted in the entire population, thus showing fixation of such mutations in the population (i.e., indicating it may have been adaptive). The outdegree and indegree are topological properties of genes in a gene regulatory network indicating, respectively, how many genes they influence and by how many they are influenced. In each subfigure genes that are expressed in all three evolutionary targets are shown as blue symbols and for each run different symbols are used. These genes are most likely to become evolutionary sensors and indeed show such behavior.
(0.04 MB PDF)

Figure S4 A Network Switching Attractor. Genes are represented by nodes, labeled as (identification tag: expression threshold), colored blue if active, or a blue outline if they are active in the opposite attractor. Activating interactions are solid edges, inhibiting ones are dashed. An insertion of gene 6 changed the expression state of 7 genes. The genes 1 and 9, depicted by a dashed blue ellipse, have not changed their expression yet, though they should be activated in order to obtain maximal fitness. Compared to Figure 6, the networks have gained interactions. For visibility, both networks were pruned for interactions originating from genes that are always silent and for parallel interactions that cancel out.
(0.11 MB PDF)
