# Supplementary material for: Evolution of Evolvability in Gene Regulatory Networks
Source: PLoS Comput Biol. 2008 Jul 11;4(7):e1000112. doi: 10.1371/journal.pcbi.1000112 (PMC2432032; doi:10.1371/journal.pcbi.1000112)
Supplement: Text S1 — Parameter Dependencies, Mutation Rates, and Environmental Rate of Change (0.02 MB PDF) [file pcbi.1000112.s001.pdf]

Supporting Information

Parameter Dependencies

In order to assess the dependencies of our results on the model parameters, we varied several key parameters such as the mutation rates and the rate of alternating the evolutionary targets.

Mutation Rates

We increased and decreased all mutation rates by a factor of 2.5 with qualitatively equal results. If we raised the rates by a factor of 10, we observed evolution of evolvability, but no evolutionary sensors. Decreasing the rates further than 2.5 resulted in a population that adapted less to environmental changes. Due to the fact that the large majority of mutations is neutral, the proper advantageous mutation often just did not occur. Reducing the rates a factor of ten gave an averaging solution. The individuals tended to integrate over the two environments.

Next we investigated the ratio of gene to binding site mutation rates. In our set of 15 runs the number of events on genes was similar to these on binding sites. We increased binding sites rates 5 times and found that the influence of the evolutionary sensor diminished. In other words, the events on binding sites began to outweigh those on genes. In an extreme case we set binding site rates equal to gene mutation rates. The networks showed no topological adaptation and adapted via binding site mutations (data not shown).

Environmental Rate of Change

In analogy to the mutation rate survey we increased the evolutionary target switching rate. For simplicity we performed these simulations in a periodically changing environment rather than a Poisson one. If we started a run with an evolved population and a fast environment (period = 1000 time steps, compared to the typical run: $1/\lambda = 3333$), the evolvability was maintained. In fact, due to the fast switching, the indirect selection pressure on keeping evolvability was higher and the population showed less neutral drift. Interestingly it was possible to evolve evolvability in this fast setting. However often the process ended in a suboptimal solution. A gene that needed to switch expression from one target to the other was recruited as the evolutionary sensor.

Summary

If we summarize the collection of all performed simulations, we have 31/65 simulations showing faster adaptations and an evolutionary sensor gene, 24/65 show only faster adaptations, but not a clear signature of an evolutionary sensor and in 10/65 runs the population failed to evolve evolvability. Note that we have included all parameter settings in these counts.
