# Supplementary material for: Evolution of Evolvability in Gene Regulatory Networks
Source: PLoS Comput Biol. 2008 Jul 11;4(7):e1000112. doi: 10.1371/journal.pcbi.1000112 (PMC2432032; doi:10.1371/journal.pcbi.1000112)
Supplement: Figure S1 — For each of the 20 different types, the average copy number in the population is plotted through time. There are two clear ESs visible in this run. From t ≈ 0.5 · 105 to 2.5 · 105 gene 16 is the ES, and from t = 3 · 105 to 6 · 105 gene 6 is the sensor. With the exception of gene 1, most of the other genes do not show large fluctuations in the long term. This run is number ten in Figure 10E (first before last). (0.19 MB PDF) [file pcbi.1000112.s002.pdf]

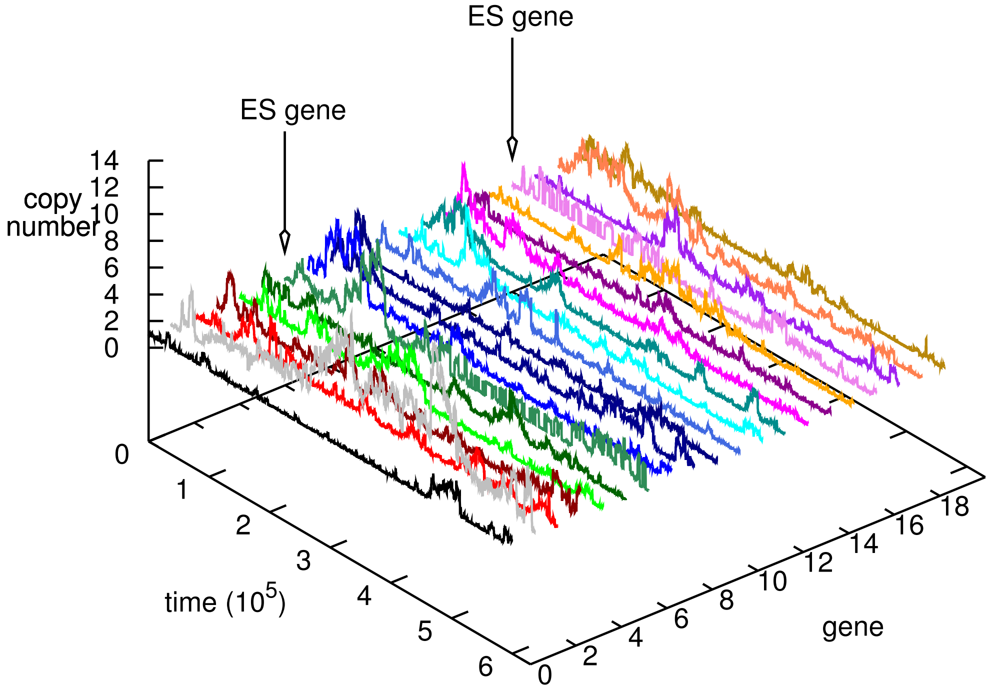

Figure S1: For each of the 20 different types the average copy number in the population is plotted through time. There are two clear ESs visible in this run. From t $\approx 0.5 \cdot 10^5$ to $2.5 \cdot 10^5$ gene 16 is the ES, and from t $= 3 \cdot 10^5$ to $6 \cdot 10^5$ gene 6 is the sensor. With the exception of gene 1 most of the other genes do not show large fluctuations in the long term. This run is number ten in Figure 10E (first before last).
