# Supplementary material for: Evolution of Evolvability in Gene Regulatory Networks
Source: PLoS Comput Biol. 2008 Jul 11;4(7):e1000112. doi: 10.1371/journal.pcbi.1000112 (PMC2432032; doi:10.1371/journal.pcbi.1000112)
Supplement: Figure S2 — For each of the 20 different types, the average copy number in the population is plotted through time. In contrast to Figure S1, we observe more fluctuations in copy numbers. Gene 18 is the ES, and for most of the run its copy number alternates between 1 and 3, or 2 and 4, which results in a fuzzier signal. Still, the gene is responsible for the adaptation. After t ≈ 5 · 105, gene 18 shows the clear-cut behavior of an ES as we have seen in Figure 4 and Figure S1. This run is number nine in Figure 10E (second before last). (0.20 MB PDF) [file pcbi.1000112.s003.pdf]

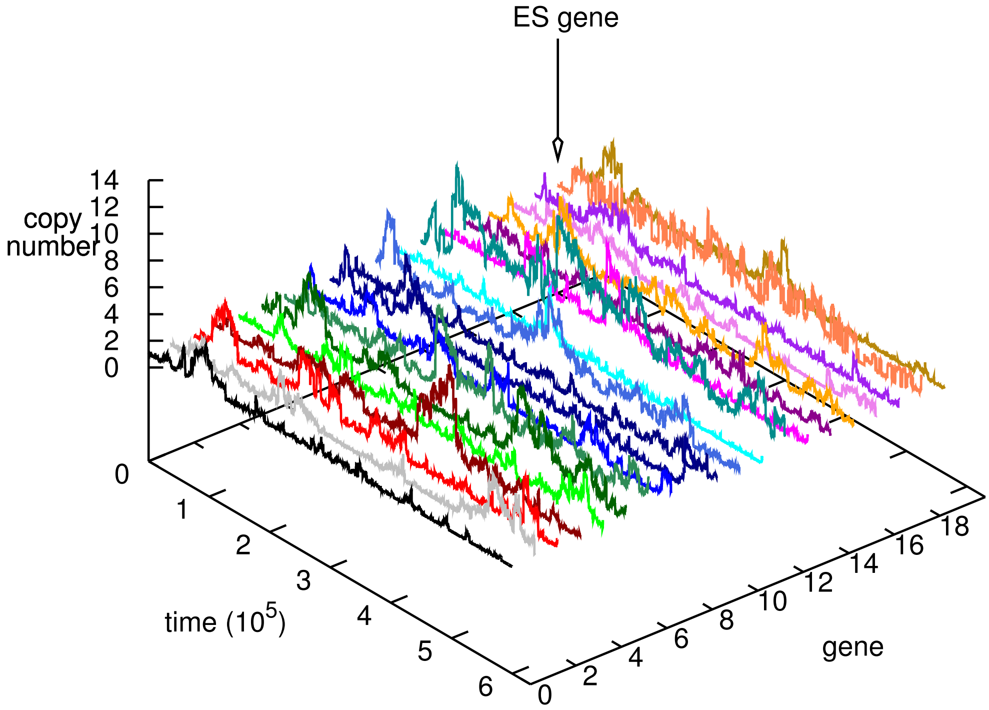

Figure S2: For each of the 20 different types the average copy number in the population is plotted through time. In contrast to Figure S1, we observe more fluctuations in copy numbers. Gene 18 is the ES, and for most of the run its copy number alternates between 1 and 3, or 2 and 4, which results in a fuzzier signal. Still the gene is responsible for the adaptation. After $t \approx 5 \cdot 10^5$ gene 18 shows the clear-cut behavior of an ES as we have seen in Figure 4 and Figure S1. This run is number nine in Figure 10E (second before last).
