# Supplementary material for: Evolution of Evolvability in Gene Regulatory Networks
Source: PLoS Comput Biol. 2008 Jul 11;4(7):e1000112. doi: 10.1371/journal.pcbi.1000112 (PMC2432032; doi:10.1371/journal.pcbi.1000112)
Supplement: Figure S3 — Scatter Plots of Gene Properties for the Case of Three Evolutionary Targets. All data points are population averages per gene type. See Model for details. (A–D) Plotted are the initial (A,C) and evolved distributions (B,D) of accumulated copy number change against outdegrees (A,B) and indegrees (C,D) of each gene. The accumulated copy number change (cp number) is a measure for how often a gene is duplicated or deleted in the entire population, thus showing fixation of such mutations in the population (i.e., indicating it may have been adaptive). The outdegree and indegree are topological properties of genes in a gene regulatory network indicating, respectively, how many genes they influence and by how many they are influenced. In each subfigure genes that are expressed in all three evolutionary targets are shown as blue symbols and for each run different symbols are used. These genes are most likely to become evolutionary sensors and indeed show such behavior. (0.04 MB PDF) [file pcbi.1000112.s004.pdf]

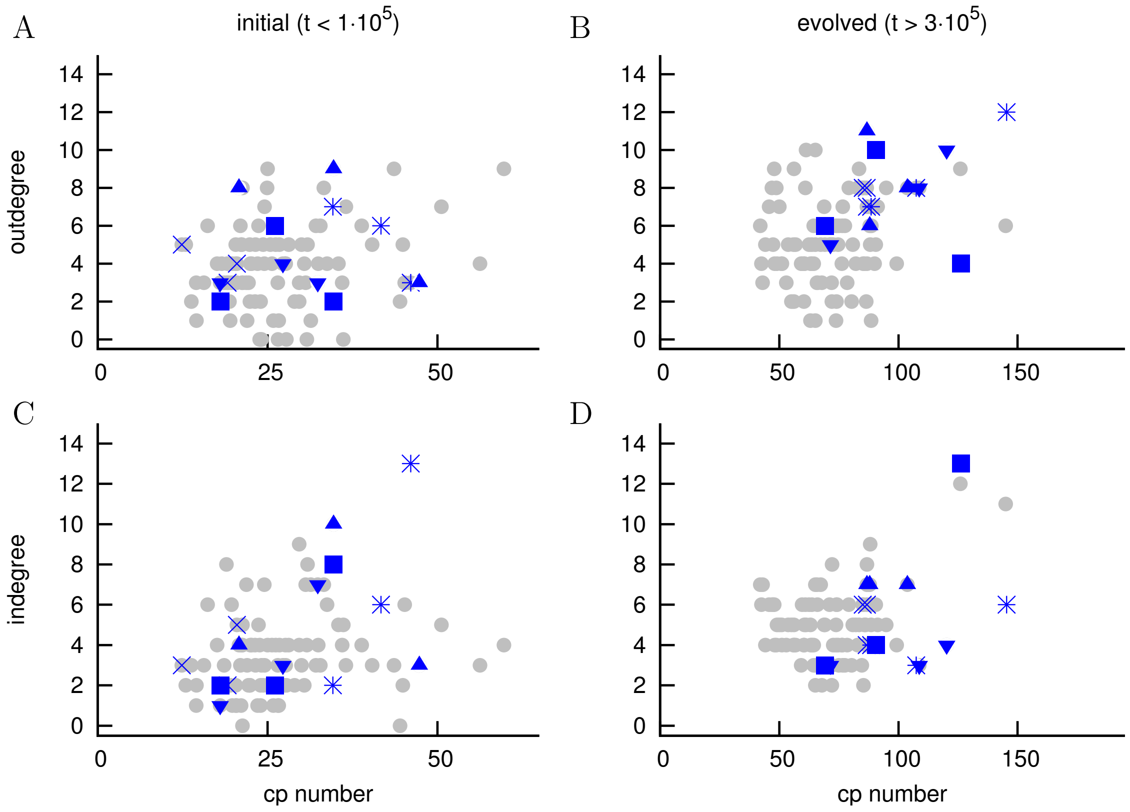


Figure S3: Scatter plots of gene properties for the case of three evolutionary targets. All data points are population averages per gene type. See Methods for details. A–D. Plotted are the initial (A, C) and evolved distributions (B, D) of accumulated copy number change against outdegrees (A, B) and indegrees (C, D) of each gene. The accumulated copy number change (cp number) is a measure for how often a gene is duplicated or deleted in the entire population, thus showing fixation of such mutations in the population (i.e. indicating it may have been adaptive). The outdegree and indegree are topological properties of genes in a gene regulatory network indicating respectively how many genes they influence and by how many they are influenced. In each subfigure genes that are expressed in all three evolutionary targets are shown as *blue* symbols and for each run different symbols are used. These genes are most likely to become evolutionary sensors, and indeed show such behavior.
