# Supplementary material for: Evolution of Evolvability in Gene Regulatory Networks
Source: PLoS Comput Biol. 2008 Jul 11;4(7):e1000112. doi: 10.1371/journal.pcbi.1000112 (PMC2432032; doi:10.1371/journal.pcbi.1000112)
Supplement: Figure S4 — A Network Switching Attractor. Genes are represented by nodes, labeled as (identification tag: expression threshold), colored blue if active, or a blue outline if they are active in the opposite attractor. Activating interactions are solid edges, inhibiting ones are dashed. An insertion of gene 6 changed the expression state of 7 genes. The genes 1 and 9, depicted by a dashed blue ellipse, have not changed their expression yet, though they should be activated in order to obtain maximal fitness. Compared to Figure 6, the networks have gained interactions. For visibility, both networks were pruned for interactions originating from genes that are always silent and for parallel interactions that cancel out. (0.11 MB PDF) [file pcbi.1000112.s005.pdf]

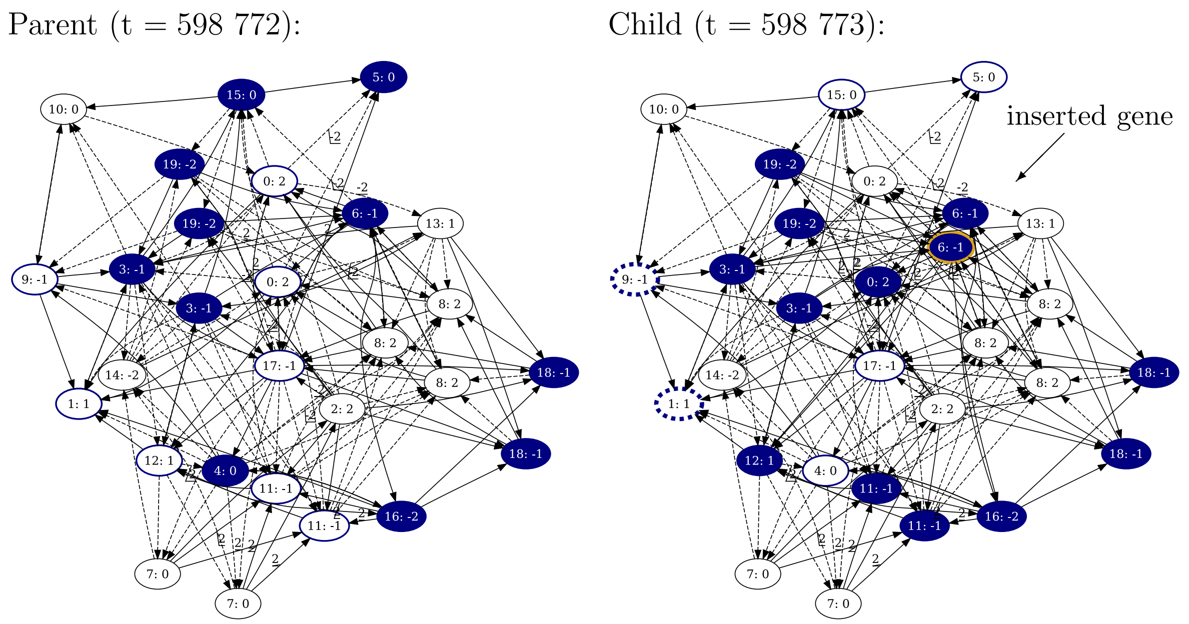

Figure S4: A network switching attractor. Genes are represented by nodes, labeled as (identification tag: expression threshold), colored blue if active, or a blue outline if they are active in the opposite attractor. Activating interactions are solid edges, inhibiting ones are dashed. An insertion of gene 6 changed the expression state of 7 genes. The genes 1 and 9, depicted by a dashed blue ellipse, have not changed their expression yet, though they should be activated in order to obtain maximal fitness. Compared to Figure 6, the networks have gained interactions. For visibility, both networks were pruned for interactions originating from genes that are always silent and for parallel interactions that cancel out.
